# Moonlighting Proteins: The Case of the Hexokinases

**DOI:** 10.3389/fmolb.2021.701975

**Published:** 2021-06-09

**Authors:** Carolina Rodríguez-Saavedra, Luis Enrique Morgado-Martínez, Andrés Burgos-Palacios, Beatriz King-Díaz, Montserrat López-Coria, Sobeida Sánchez-Nieto

**Affiliations:** Laboratorio de Transporte y Percepción de Azúcares en Plantas, Departamento de Bioquímica, Facultad de Química, Universidad Nacional Autónoma de México, Mexico City, Mexico

**Keywords:** hexokinase, moonlighting function, sensor proteins, glycolytic moonlighting proteins, hexokinase regulation mechanisms

## Abstract

Moonlighting proteins are defined as proteins with two or more functions that are unrelated and independent to each other, so that inactivation of one of them should not affect the second one and vice versa. Intriguingly, all the glycolytic enzymes are described as moonlighting proteins in some organisms. Hexokinase (HXK) is a critical enzyme in the glycolytic pathway and displays a wide range of functions in different organisms such as fungi, parasites, mammals, and plants. This review discusses HXKs moonlighting functions in depth since they have a profound impact on the responses to nutritional, environmental, and disease challenges. HXKs’ activities can be as diverse as performing metabolic activities, as a gene repressor complexing with other proteins, as protein kinase, as immune receptor and regulating processes like autophagy, programmed cell death or immune system responses. However, most of those functions are particular for some organisms while the most common moonlighting HXK function in several kingdoms is being a glucose sensor. In this review, we also analyze how different regulation mechanisms cause HXK to change its subcellular localization, oligomeric or conformational state, the response to substrate and product concentration, and its interactions with membrane, proteins, or RNA, all of which might impact the HXK moonlighting functions.

## Introduction

Many proteins can perform different independent functions, which are known as “moonlighting proteins” (Jeffery, 1999). This term has been well-accepted to designate proteins with other “jobs” in the cells (Huberts and van der Klei, 2010). Although this characteristic may be seen as multifunctionality, moonlighting proteins are defined as proteins with two or more functions that are unrelated to each other. Proteins whose different functions are due to gene fusion, splice variants, or those that are distributed in separate subcellular compartments but perform the same role in each of them are excluded (Jeffery, 1999; Gancedo and Flores. 2008).

The first reports describing moonlighting proteins were published in the 1980s. Piatigorsky and Wistow (1989) noticed that some crystallins (structural proteins in the lens of vertebrate eyes) were metabolic enzymes: duck ε-crystallin was actually lactate dehydrogenase B4 (Hendriks et al., 1988), and τ-crystallin was α-enolase (Wistow et al., 1988). The enzymes’ ability to function as crystallin proteins is based on their thermodynamic stability and their ability to concentrate at high levels without precipitating. Additionally, the enzymes’ metabolic role in the lens tissue is unlikely, having a structural role instead, indicating that both proteins’ function depends on cell type (Wistow and Piatigorsky, 1988).

Up until 2015, around 300 moonlighting proteins had been characterized in many different organisms performing equally diverse functions (Mani et al., 2015), which can be found in databases such as MoonProt (http://www.moonlightingproteins.org; Mani et al., 2015) and MultitaskProtDB (http://wallace.uab.es/multitask/; Hernández et al., 2014). From the moonlighting proteins that have been experimentally characterized, there are several bioinformatic approaches to predict whether a protein can moonlight (Hernández et al., 2015).

A crucial feature to designate a protein as moonlighting is that its functions must be independent, so that inactivation of one of them should not affect the second one and vice versa (Huberts and van der Klei, 2010). Moonlighting activities can be two different enzymatic activities, combine a catalytic function with a non-catalytic activity, or even two non-catalytic functions (Rad et al., 2018). Moonlighting proteins are distributed in all living beings, and a wide range of examples have been described in yeasts (Gancedo and Flores. 2008), mammals (Sriram et al., 2005), protozoa (Collinridge et al., 2010), bacteria (Arnér and Holmgren 2000) and plants (Aguilera-Alvarado and Sánchez-Nieto., 2017). Most moonlighting proteins were initially described for a well-defined function in a biochemical pathway, like human aconitase (Kennedy et al., 1992) or *Trypanosoma cruzi* mevalonate kinase (Goldstein and Brown, 1990); or for participating in essential physiological processes, as reactive oxygen species (ROS)-defense mechanisms, like superoxide dismutase 1 (Sod1) in *Saccharomyces cerevisiae* (Tsang et al., 2014); or for assistance in protein folding or assembly of macromolecular complexes, like bacterial molecular chaperones (Hagemann et al., 2017). In general, proteins with the ability to moonlight can be a remarkably diverse group of proteins. Their moonlighting functions are different and very diverse depending on the organism. Considering the examples mentioned above, human aconitase moonlights as an intracellular sensor of iron levels (Artymiuk and Green, 2006); *T. cruzi* mevalonate kinase has an additional function as binding factor to cell host membranes (Bahia, 2017); *S. cerevisiae* Sod 1 acts as a transcription factor of genes related to oxidative stress response (Gancedo et al., 2016) and bacterial molecular chaperones moonlight as host cell adhesion factors (Henderson and Martin, 2011). The switching between one moonlighting activity to another can vary as a consequence of changes in subcellular localization (Sun et al., 2018), oligomeric or conformational state (Vega et al., 2016), ligands, substrates, cofactors or product concentration (Artymiuk and Green, 2006), interactions with DNA or RNA (Jeffery, 1999), membrane interactions (Jeffery, 2009), cell types (Wistow and Piatigorsky, 1988) and even in response to environmental factors (Copley, 2014).

This review addresses the diverse moonlighting functions ascribed to hexokinases (HXKs) in different kingdoms. HXK is a key enzyme in the metabolism and belongs to the most recognized moonlighting protein group: the glycolytic enzymes. This review also focuses on the consequences of the regulation of the HXK by changes in its subcellular location, oligomeric conformation, RNA and membrane interaction.

## Moonlighting Proteins in the Glycolytic Pathway

According to Singh and Bhalla (Singh and Bhalla, 2020), proteins constitutively expressed, as those belonging to metabolic pathways, are better candidates to evolving moonlighting functions as they have a higher chance of interacting in a transient or a stable manner with different molecules, such as RNA, DNA or even other proteins, hence, leading to a benefit. This is supported by the REM (RNA-enzyme-metabolite) hypothesis, which postulates the existence of regulatory networks between gene expression and intermediary metabolism mediated by moonlighting RNA-binding metabolic enzymes (Hentze and Preiss, 2010). Examples of enzymes leading to the REM hypothesis are hexokinase (HXK), aldolase (ALDO), glyceraldehyde-3-phosphate dehydrogenase (GAPDH), enolase (ENO) and pyruvate kinase (PK) in HeLa, HEK293 and mouse embryonic stem cells (Castello et al., 2015). Furthermore, HXK, Phosphofructokinase (PFK), ALDO, GAPDH, Phosphoglycerate Kinase (PGK), Phosphoglycerate Mutase (PGM), ENO and PK, have been found inside the nucleus of *Saccharomyces cerevisiae* and cancer and muscle cells from *Homo sapiens* (Kim and Dang, 2005; Boukouris et al., 2016). At this location, these proteins carry out a wide range of functions either in complex with DNA or in complex with other proteins, some of which are listed in Table 1 (for further information see Boukouris et al., 2016 and references within). It is not clear if the main purpose of these proteins in the nucleus is providing ATP and NADH, yet it is thought that their unusual location may be the result of evolutionary pressure. Around the REM hypothesis, several questions arise: are glycolytic enzymes capable of binding directly to RNA, or do they have to interact with other partners to do so? Interestingly, many of them share moonlighting functions as adhesion or transcriptional factors in different organisms: how can certain moonlighting functions be conserved throughout species? And, regarding this, it is also intriguing that since glycolysis is an essential and conserved metabolic pathway, it is remarkable that every of its enzymes play very different moonlighting roles, so how is it that they were selected to carry out different functions in different cell locations, under different environmental conditions and/or different stages of development?

**TABLE 1:** Moonlighting function of nine glycolytic enzymes.

Enzyme	Canonical function	Moonlighting function	Organism	References
Glucose-6-phosphate isomerase	Glucose-6-phosphate 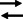 fructose-6-phosphate	Adhesion protein	*Lactobacillus crispatus*	Kainulainen et al. (2012)
		Autocrine motility factor	*Homo sapiens*	Wantanabe et al. (1996)
		Neuroleukin	*Homo sapiens*	Sun et al. (1999)
		Virulence	*Xanthomonas oryzae*	Tsuge et al. (2004)
Phosphofructokinase	Fructose-6-phosphate 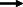 fructose-1,6-bisphosphate	Autophagy regulator	*Pichia pastoris*	Yuan et al. (1997)
		Part of RNA degradosome complex	*Bacillus subtilis*	Commichau et al. (2009)
		Transcriptional regulator	*Drosophila melanogaster*	Enzo et al. (2015)
Aldolase	Fructose-1,6-bisphosphate 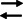 glyceraldehyde-3-phosphate + dihydroxyacetone phosphate	Actin dynamics controller	*Oryctolagus cuniculus*	Ritterson-Lew and Tolan (2013)
		GLUT4 regulator	*Homo sapiens*	Kao et al. (1999)
		Involved in pathogen-host interaction	*Candida albicans*	Crowe et al. (2003)
			*Plasmodium berghei*	Bosch et al. (2007)
			*Streptococcus pneumoniae*	Blau et al. (2007)
			*Neisseria meningitidis*	Tunio et al. (2010b)
			*Paracoccidioides* spp.	Marcos et al. (2014)
		Transcriptional regulator	*Mus musculus*	Kiri and Goldspink (2002)
			*Francisella tularensis*	Ziveri et al. (2017)
		V-ATPase assembly and activity mediator	*Mus musculus*	Lu et al. (2001)
		Zona pellucida recognition	*Homo sapiens*	Petit et al. (2013)
Triosephosphate isomerase	Dihydroxyacetone phosphate 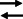 glyceraldehyde-3-phosphate	Endoglucanase	*Pyrococcus furiosus*	Sharma and Guptasarma (2017)
		Pathogen establishment in host	*Paracoccidioides brasiliensis*	Pereira et al. (2007)
			*Streptococcus anginosus*	Kinnby et al. (2008)
			*Streptococcus oralis*	Kinnby et al. (2008)
			*Lactobacillus plantarum*	Ramiah et al. (2008)
			*Staphylococcus aureus*	Ikeda and Ichikawa (2014)
		Zona pellucida recognition	*Homo sapiens*	Petit et al. (2013)
Glyceraldehyde-3-phosphate dehydrogenase	Glyceraldehyde-3-phosphate 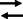 1,3-bisphosphoglycerate	Acts as SSBP	*Bos taurus*	Grosse et al. (1986)
		Apoptosis regulator	*Mus musculus*	Dastoor and Dreyer (2001)
			*Rattus novergicus*	Hara et al. (2005)
			*Homo sapiens*	Yego and Mohr (2010)
		Autophagy regulator	*Arabidopsis thaliana*	Henry et al. (2015)
			*Nicotiana benthamiana*	Han et al. (2015)
		Extracellular polysaccharide synthesis	*Xanthomonas campestris*	Lu et al. (2009)
		Flagellar protein	*Trypanosoma brucei*	Brown et al. (2014)
		Immunomodulator	*Streptococcus agalatactiae*	Madureira et al. (2007)
			*Haemonchus contortus*	Sahoo et al. (2013)
		Pathogen establishment in host	Group a streptococci	Pancholi and Fischetti (1992)
			*Lactobacillus crispatus*	Hurmalainen et al. (2007)
			*Escherichia coli*	Egea et al. (2007)
			*Schistosoma bovis*	Ramajo-Hernández et al. (2007)
			*Bacillus anthracis*	Matta et al. (2010)
			*Neisseria meningitidis*	Tunio et al. (2010a)
			*Paracoccidioides spp*	Marcos et al. (2014)
			*Erysipelothrix rhusiopathiae*	Zhu et al. (2017)
		Transferrin receptor	Streptococci	Modun et al. (2000)
		Transcriptional regulator	*Homo sapiens*	Zheng et al. (2003)
			*Mus musculus*	Ikeda et al. (2012)
			*Arabidopsis thaliana*	Kim et al. (2020)
		DNA glycosylase	*Homo sapiens*	Meyer-Siegler et al. (1991)
		Zona pellucida recognition	*Homo sapiens*	Petit et al. (2013)
Phosphoglycerate kinase	1,3-bisphosphoglycerate 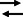 3-phosphoglycerate	Binds to polymerase increasing DNA synthesis	*Homo sapiens*	Popanda et al. (1998)
		Flagellar protein	*Mus musculus*	Sawyer et al. (2008)
			*Trypanosoma brucei*	Brown et al. (2014)
		Pathogen establishment in host	Group B streptococci	Boone et al. (2011)
		Plasmin reductase	*Homo sapiens*	Lay et al. (2000)
		Transcriptional regulator	*Homo sapiens*	Shetty et al. (2004)
Phosphoglycerate mutase	3-phosphoglycerate 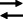 2-Phosphoglycerate	Involved in bacterial adhesion to host	*Candida albicans*	Crowe et al. (2003)
			*Bifidobacterium lactis*	Candela et al. (2007)
Enolase	2-phosphoglycerate 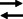 phosphoenolpyruvate	Immunosuppressing protein	*Streptococcus sobrinus*	Veiga-Malta et al. (2004)
			*Steinernema glaseri*	Liu et al. (2012)
		Involved in encystation mechanism	*Entamoeba invadens*	Segovia-Gamboa et al. (2010)
			*Naegleria fowleri*	Chávez-Munguía et al. (2011)
		Neurotrophic and neuroprotective factor	*Rattus novergicus*	Hattori et al. (1995)
		Pathogen establishment in host	*Pneumocystis carinii*	Fox and Smulian (2001)
			*Candida albicans*	Jong et al. (2003)
			*Streptococcus mutans*	Jones and Holt (2007)
			*Leishmania mexicana*	Vanegas et al. (2007)
			*Bacillus anthracis*	Agarwal et al. (2008)
			*Aeromonas hydrophila*	Sha et al. (2009)
			*Paracoccidioides brasiliensis*	Nogueira et al. (2010)
		Tau lens crystalline	*Pseudemys scripta*	Wistow et al. (1988)
		Transcriptional regulator	*Homo sapiens*	Subramanian and Miller (2000)
			*Toxoplasma gondii*	Mouveaux et al. (2014)
Pyruvate kinase	Phosphoenolpyruvate 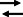 Pyruvate	Bacterial adhesion	*Lactococcus lactis*	Katakura et al. (2010)
		Hormone binding protein	*Rattus novergicus*	Parkinson et al. (1991)
		Protein kinase	*Homo sapiens*	Gupta and Bamezai (2010)
			*Saccharomyces cerevisiae*	Li et al. (2015)
		Transcriptional regulator	*Homo sapiens*	Gao et al. (2012)
		Zona pellucida recognition	*Homo sapiens*	Petit et al. (2013)

In bacteria, the fact that most of the glycolytic enzymes help the microorganisms colonize or establish on host’s cells has led to hypothesize that the reason why glycolytic enzymes moonlight is because as both bacterial and human glycolytic enzymes present a high degree of conservation, the host immune system will not elicit antibodies against the pathogens’ glycolytic enzymes (Franco-Serrano et al., 2018). According to Wang et al., 2015, autoimmune diseases affect from 3—5% of the world population, with autoimmune thyroid disease and type one diabetes being the two most common ones. Amela et al., 2007 (a previous paper published by the same group as Franco-Serrano, et al., 2018), showed that only 1% of 2,175 proteins analyzed contain a sequence that can be found in both pathogen and host, suggesting that this might be a mechanism for a pathogen to avoid the host’s immune system. In spite of this, it has been found that it is possible to generate autoantibodies raised against glycolytic enzymes. Antibodies against TPI (Vermeulen et al., 2011), ALDO (Magrys et al., 2007; Goëb et al., 2009; Vermeulen et al., 2011), GAPDH (Adamus et al., 2011), PGK (Goëb et al., 2009), PGM (Kimura et al., 2010; Vermeulen et al., 2011) and ENO (Terrier et al., 2007; Goëb et al., 2009; Adamus et al., 2011; Vermeulen et al., 2011; Maccallini et al., 2018) are linked to the development of retinopathy, rheumatoid arthritis, autoimmune hepatitis, inflammatory bowel disease and Lyme disease. The high expression of the glycolytic proteins in the diseases mentioned before, together with frequent infections and chronic inflammation, and the high extracellular exposure of some of these protein may promote the production of autoantibodies (Adamus, 2017), suggest that “mimicking” glycolytic enzymes in order to establish on a host, might not be the best mechanism.

## Hexokinase

Hexokinase was first described by Otto Meyerhof who demonstrated the stimulatory effect of a baker’s yeast extract on the fermentation of hexoses (Meyerhof, 1927). Almost a decade later, von Euler and Adler (1935) and Meyerhof (1935), determined that this enzyme catalyzes the phosphorylation of hexoses using ATP as a phosphoryl donor. Later, Colowick and Kalckar (1943) showed that only the γ-phosphate group was transferred from ATP to the hexoses. By 1953, the first plant hexokinase (from *Triticum aestivum*) was described (Saltman, 1953), giving enough evidence to state that hexokinases can be found in microorganisms, animals, and plants.

The existence of different hexokinase isoenzymes was first found in *Saccharomyces cerevisiae* in 1961 (Tayser and Colowick, 1961). In the same decade (1960s) mammal hexokinase isoenzymes were reported in rat liver (González et al., 1964), human and dogs (Brown et al., 1967). At last, Jang et al. (1997) demonstrated that there are also hexokinase isozymes in plants.

**FIGURE 1:**
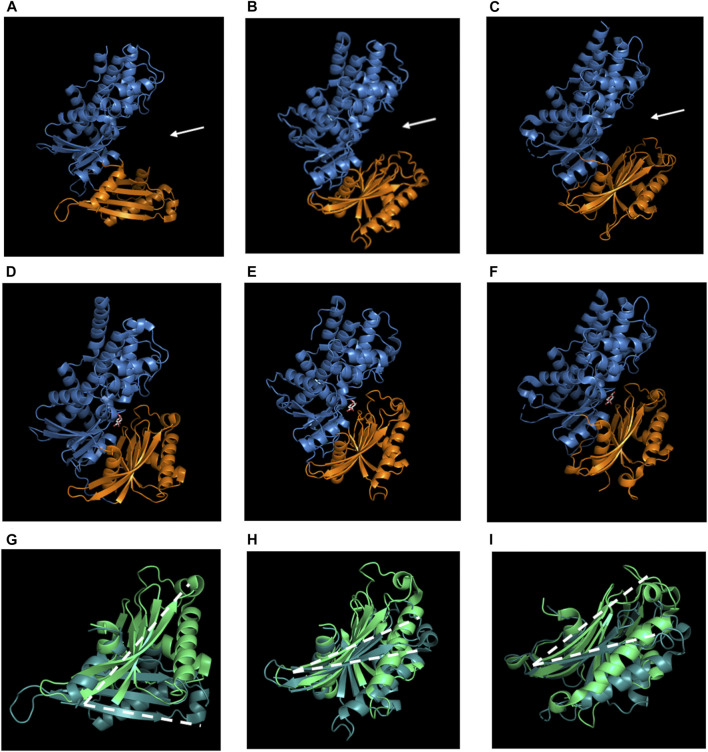
Structural overview of hexokinases. **A, D and G** Human hexokinase IV (HXKIV), **B, E and H** *Kluyveromyces lactis* hexokinase 1 (KlHXK1) and **C, F and I** *A. thaliana* hexokinase 1 (AtHXK1). **A, B and C** are the Apo-form of the HXKs, **D, E and F** are the glucose-bound form and **G, H and I** are the comparison between the small domain of the Apo-HXK (dark green) and the Glc-HXK (light green). **A, B and C** exhibit a similar tertiary structure; they display a large (blue) and a small (orange) domain. Between both domains, there is a cleft (white arrows) where the active site is located. In **D, E and F** the small domain approaches to the large one to interact with glucose and carry out the catalysis and although domain approaching occurs in all of the three cases mentioned, it does not occur to the same extent; **G, H and I** show a comparison between the small domain of the Apo-HXK (dark green) vs. the Glc-HXK (light green). The white dotted line represents the angle of rotation of the small domain toward the big one. Glucose is represented as white sticks in **D, E and F**. Images of 1V4T (Kamata et al., 2004), 3O80 (Kuettner et al., 2010), 4QS8 (Feng et al., 2015), 3IDH (Petit et al., 2011), 3O8M (Kuettner et al., 2010) and 4QS7 (Feng et al., 2015) were created using PyMOL. The PyMOL Molecular Graphics System. Version 2.3 Schrödinger, LLC.

Hexokinases have similar structures between different organisms (Figure 1); they have two domains, the large domain has an oval shape that may or may not contain a targeting location sequence on the N-terminus, and a small domain with a three-layer architecture (Kamata et al., 2004; Feng et al., 2015). It should be clarified that in this review and in previous papers related to hexokinases’ structure, the term “domain” is used to refer to a subregion of a protein that is autonomous in the sense that it possesses all the characteristics of a complete globular protein, as stated by Schulz and Schirmer (1979). Not only do hexokinases have a similar tertiary structure, but also a similar number of secondary structures, i.e. human hexokinase IV, HXKIV, has 15 α-helices (Figure 1A), whilst *Klyuveromyces lactis* hexokinase 1, KlHXK1 (Figure 1B), has 13 and *Arabidopsis thaliana* hexokinase 1, AtHXK1 (Figure 1C), has 14; AtHXK1 has 12 β-strands and HXKIV has 13 and KlHXK1 has 18. Another characteristic shared by hexokinases is that upon glucose binding, their domains approach to each other to a different extent, adopting the catalytically active closed conformation (Figures 1D–F) (Bennett and Steitz, 1980; Kamata et al., 2004; Petit et al., 2011; He et al., 2019). Besides those structural similarities, AtHXK1 is not only a glucose phosphorylating enzyme but also a glucose sensor protein, so deeper analysis needs to be done to find the features that are important for HXKs to moonlighting.

## Function of the Hexokinases as Glucose-Sensor is Conserved

Out of the non-canonical functions of the hexokinase, probably the one that has been described in a larger number of organisms is the glucose-sensing ability (Table 2). Hexokinases able to act as glucose sensors have been described in mammals and several other vertebrates, including fishes (Matschinsky, 1993; Irwin and Tan, 2014; Heneberg, 2019), *Saccharomyces cerevisiae* (Rolland et al., 2002), *Arabidopsis thaliana* (Moore et al., 2003), *Solanum tuberosum* (Veramendi et al., 2002), *Oryza sativa* (Cho et al., 2009; Kim et al., 2016), *Nicotiana tabacum* (Kim et al., 2013b), *Chromochloris zofingiensis* (Roth et al., 2019), *Klebsormidium nitens* (Ulfstedt et al., 2018) and *Synechocystis sp* (Ryu et al., 2008). Despite being well-known glucose-sensing proteins, the precise mechanism responsible for this moonlighting activity is not clearly understood yet. Several recent advances in the single molecule enzymology and the development of new computational analysis are giving insight on the enzymes’ behavior that could led to explain its moonlighting ability, for instance, mammal HXKIV (GCK), it has been suggested that its glucose-sensor activity is due to its low glucose affinity (*K*_*m*_ ≈ 10 mM, Bustos and Iglesias, 2000; Larion and Miller, 2012) together with its kinetic cooperativity. The discovery of different enzyme conformers after the first enzyme cycle, explains the cooperativity that enables an adequate response to low glucose concentration and avoid depleting blood glucose levels (Larion et al., 2015; Šimčíková et al., 2017). A similar explanation might explain why ScHXK2 suffers a conformational change in response to cytoplasmic glucose levels that allows the enzyme to form the repressor complex that leads to the repression of *SUC2* gene (Vega et al., 2016). In some cases, determining the three-dimensional structure has shed light on elucidating how a protein can have two different functions (Jeffery, 2004). Unfortunately, as far as hexokinases are concerned, this task is limited because of the few amount of crystallized glucose sensing hexokinases, combined with the absence of non-glucose sensing HXK structures to make a comparison. In fact, from the organisms listed previously, it has only been possible to crystalize hexokinase II from *S. cerevisiae* (Kuser et al., 2000), hexokinase IV from *Homo sapiens* (Kamata et al., 2004), hexokinase 1 from *A. thaliana* (AtHXK1; Feng et al., 2015) and hexokinase 6 from *O. sativa* (OsHXK6; He et al., 2019).

**TABLE 2:** Confirmed non-canonical activities of hexokinases.

Moonlighting function	Organism	References
Apoptosis regulator	*Homo sapiens*	Pastorino et al. (2002)
	*Nicotiana tabacum*	Godbole et al. (2013)
	*Nicotiana benthamiana*	Kim et al. (2006)
Autophagy regulator	*Homo sapiens*	Kundu (2014)
Intracellular glucose sensor	*Arabidopsis thaliana*	Moore et al. (2003)
	*Chromochloris zofingiensis*	Roth et al. (2019)
	*Klebsormodium nitens*	Ulfstedt et al. (2018)
	*Oryza sativa*	Cho et al. (2009), Kim et al. (2016)
	*Nicotiana tabacum*	Kim et al. (2013b)
	*Saccharomyces cerevisiae*	Johnston (1999)
	*Solanum tuberosum*	Veramendi et al. (2002)
Protein kinase	*Homo sapiens*	Adams et al. (1991)
	*Malus domestica*	Hu et al. (2016), Sun et al. (2018)
	*Saccharomyces cerevisiae*	Herrero et al. (1989)
Transcriptional regulator	*Arabidopsis thaliana*	Cho et al. (2006b)
	*Candida albicans*	Laurian et al. (2019)
	*Homo sapiens*	Sheikh et al. (2018)
	*Kluyveromyces lactis*	Prior et al. (1993)
	*Oryza sativa*	Huang et al. (2015)
	*Saccharomyces cerevisiae*	Herrero et al. (1998)
	*Vitis vinifera*	Wang et al. (2017)

The crystal structure of ScHXK2, AtHXK1, and OsHXK6 show great resemblance with other non-moonlighting hexokinases: they have almost the same number of secondary structures forming two domains which are connected by hinges, and between these two domains the active site is located. When bound to glucose, one domain rotates toward the other about 20° (Anderson et al., 1979; Bennett and Steltz, 1980; Feng et al., 2015; He et al., 2019). Besides, amino acids involved in catalysis are conserved.

One of the characteristics of moonlighting proteins is that the inactivation of the canonical function does not hamper the non-canonical function or vice-versa. This was proved by crystalizing the catalytically inactive mutant AtHXK1S177A (which still retains its glucose-sensing ability according to Moore et al., 2003) complexed with glucose (Feng et al., 2015); the 3D structure revealed that both domains still approach and are still able to interact with glucose in a similar fashion to wild type hexokinase, with an overall root mean square deviation (r.m.s.d.) of 0.45 Å. Additionally, AtHXK1 forming a complex with VHA-B1 and RPT5B is known to regulate the transcription of photosynthesis-related genes when bound to glucose (Cho et al., 2006b). These two reports suggest that the AtHXK1-Glc complex could be the active conformation able to moonlight, although further work is still needed to find the contacts between the different HXK patterns.

## Hexokinases Moonlighting Functions in Diverse Life Kingdoms

### Protozoa

In *Trypanosoma brucei*, TbHK1 and TbHK2 are expressed in bloodstream-form and procyclic stage parasites in different developmental stages, and TbHK2 has been localized in the flagelum inside and outside of the glycosomes, specialized structures that contain glycolytic enzymes (Blattner et al., 1995; Michaels et al., 2006). The ability of TbHK2 to be on different compartments in the cell might help to have different functions. Additionally, recombinant TbHXK2 is inactive, but when mixed with TbHXK1 also as a recombinant protein, the heterooligomer is active with kinetic properties different from the TbHXK1 recombinant enzyme alone, suggesting a regulatory role of TbHXK1, likely a glucose sensor to regulate the glycolysis flux and ATP production (Joice et al., 2012). However, these hypotheses remain to be demonstrated, as well as to elucidate the mechanism involved in the translocation of TbHK2 from glycosomes to the flagellum, the interaction partners involved, and whether the functions of both HXKs as glucose-phosphorylating enzymes and as Hb binding protein or sugar sensor are uncoupled or not.

### Yeasts and Fungi

**FIGURE 2:**
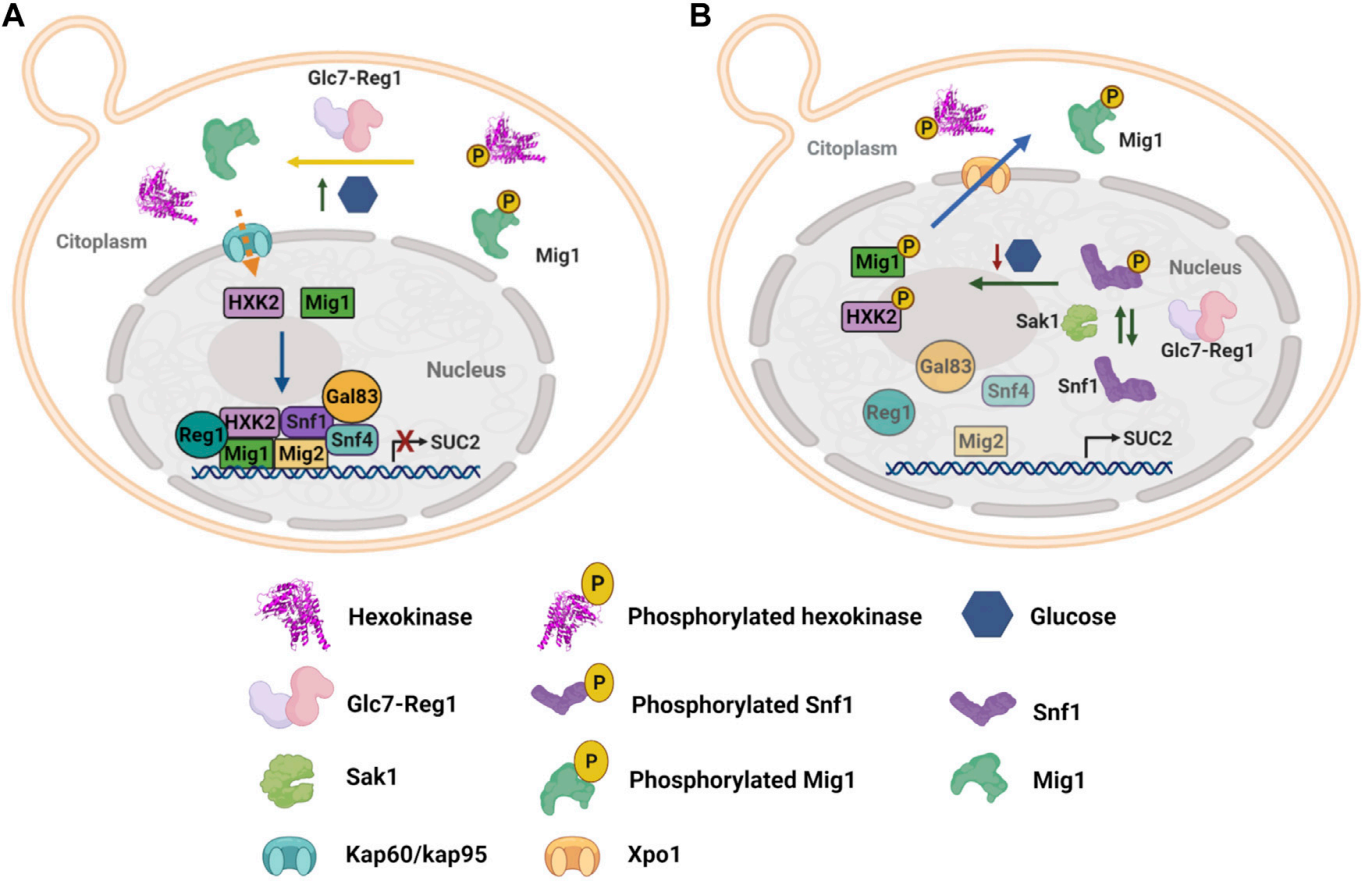
Yeast hexokinase moonlighting function and regulation. **(A)** Under high glucose conditions, ScHXK2 and Mig1 are dephosphorylated by the phosphorylated Glc7-Reg1 protein phosphatase, which in this state has a nuclear localization. Glc7-Reg1 also dephosphorylates Snf1 kinase and therefore it is maintained inactivated. Thus, at high glucose concentration, a repressor complex is formed at the *SUC2* promoter, which represses gene expression. The repressor complex consists of transcriptional repressors as Mig1 and Mig2 directly bound to the DNA; the protein Hxk2, the phosphatase Reg1-Glc7, and the three subunits of the SNF1 complex, Snf1, Snf4, and Gal83. **(B)** In low glucose conditions, Sak1 kinase activates Snf1 by phosphorylation of threonine 210. Interaction between ScHXK2 and Mig1 is abolished due to phosphorylation of both proteins by Snf1 kinase, which results in the disassembly of the *SUC2* repressor complex and the export of ScHXK2 and Mig1 to the cytoplasm, allowing the transcription of *SUC2* (Herrero et al., 1998; Randez-Gil et al., 1998; Ahuatzi et al., 2004; Ahuatzi et al., 2007; Peláez et al., 2009; Fernández-García et al.,; Peláez et al., 2012; Vega et al., 2016).

Yeasts comprise an excellent example of the glucose sensor moonlighting activity of HXKs. *S. cerevisiae* expresses two cytosolic hexokinases: ScHXK1 and ScHXK2; and one glucokinase: ScGLK (Randez-Gil et al., 1998). ScHXK2 has been extensively described for its moonlighting properties participating in both glucose catabolism and glucose catabolite repression, which has been demonstrated to be mutually uncoupled functions (Ahuatzi et al., 2004; Gancedo et al., 2014). In the glucose catabolite repression process, ScHXK2 functions as a regulator of gene transcription in the nucleus, so that under high glucose conditions ScHXK2 translocates from the cytosol to the nucleus and once inside, it interacts with C2H2 zinc-finger transcription factor Mig1 (Randez-Gil et al., 1998; Ahuatzi et al., 2004) to recruit other proteins in order to form a repressor complex at gene promoters encoding catabolic enzymes of non-fermentable sugars, such as *SUC2* gene, repressing their transcription (Figure 2A) (Herrero et al., 1998; Ahuatzi et al., 2004; Vega et al., 2016). Contrary, low glucose concentration abolish the interaction between ScHXK2 and Mig1, and the repressor complex is disassembled, thus allowing transcription of *SUC2* gene, which encodes cytoplasmic and extracellular invertases (Figure 2B) (Ahuatzi et al., 2007; Fernández-García et al.,).

Hexokinase 1 from *Yarrowia lipolytica* (YlHXK1) might also be a moonlighting protein; according to Hapeta et al., 2021, this hexokinase presents a unusual loop of 37 amino acids long that is not present in other hexokinases from the Yarrowia genus. *Y. lipolytica* cells, expressing YlHXK1 lacking this loop (YlHXK1Δloop), are unable to phosphorylate neither glucose nor fructose, apparently due to the conformational changes triggered by the loop deletion. Furthermore. by overexpressing the YlHXK1Δloop, the expression of lipases 2 and 8 (LIP2 and LIP8) is diminished. Unluckily, research on the effect that point mutations of amino acids involved in catalysis have on the expression of LIP2 and LIP8 is still needed in order to prove whether the downregulation of these lipases is due to incapability to phosphorylate glucose rather than the conformational changes of YlHXK1Δloop.

Studies on other fungi HXKs have been made by analyzing the phenotype of HXKs null mutants. *Fusarium graminearum*, the cause of *Fusarium* head blight (FHB), produces a range of mycotoxins, like type B trichothecenes, potent inhibitors of eukaryotic protein synthesis, being deoxynivalenol (DON) the main trichothecene produced having a serious impact on crops, human and animal health (Alexander et al., 2009). Null mutants of the two HXKs in *F. graminearum* showed that FgHXK1, but no FgHXK2 deletion produced significantly low levels of DON, indicating that FgHXK1 might be involved in increased DON production, and may have a possible indirect role on virulence and *F. graminearum* spread within spikes. Additionally, *fghxk1* null mutants also exhibited defects in growth, pathogenicity, conidial, and perithecia production, suggesting a role in such processes (Zhang et al., 2016). Null *hkx1* mutants in *F. verticilloides*, the causal agent of maize ear rot worldwide, were substantially less virulent and produced fivefold less fumonisin B1 (FB1) toxin than the wild-type, and the production of FB1 was restored in the complemented strain, indicating that HXK1 is required for FB1 biosynthesis. Though the exact mechanism through which HXK1 affects FB1 production is still unknown, authors suggest that carbon catabolite repressor protein, CreA, a C2H2 zinc-finger protein, may indirectly regulate FB1 biosynthesis through the regulation of sugar transporters and sugar kinases, as they found that HXK1 has four putative CreA binding sites within 500 bp upstream of the start codon (Desai et al., 2002; Kim et al., 2011). Taken together, these reports suggest that fungi HXKs may have a much broader regulatory role that remains to be explored.

### Mammals

In mammals, hexokinases present a wide range of functions, but in which it is still questionable their moonlighting ability (Dubey et al., 2016; Affourtit et al., 2018). For example, the human genome encodes five hexokinases that phosphorylate glucose using ATP as a phosphoryl donor: HXKI, HXKII, HXKIII, HXKIV, the latter also known as glucokinase (GCK), and HXK domain protein containing 1 or HXKDC1. HXKDC1 was recently found in a genome wide association study and it is implicated in glucose tolerance, especially during pregnancy. It has a high degree of similarity with HXKI and show hexokinase catalytic activity, however, it is possible that it also regulates the activity of other HXKs (Guo et al., 2015; Ludvik et al., 2016). Another glucose phosphorylating enzyme found in humans is ADPGK, this endoplasmic reticulum enzyme is quite different from the other HXKs; it uses ADP as a phosphoryl donor and shows optimal activity at high temperature (42°C) and acidic pH and seems to be implicated in the regulation of the energy metabolism and protein glycosylation due to its subcellular location (Imle et al., 2019). Each of the hexokinase isoforms expresses differentially through tissues, being HXKI the one that expresses ubiquitously, though it is mainly expressed in the brain, and therefore suggesting a mere catabolic role. HXKII is the predominant isoform in skeletal and cardiac muscle, as well as in adipocytes, though an increase in its expression occurs in cancer cells, even in tissues where its expression is negligible under health physiological conditions (Mathupala et al., 2006). HXKIII is also ubiquitously expressed, but is not predominant in any tissue, while glucokinase is found in pancreatic α- and δ-cells, adrenal gland, glucose-sensitive neurons, enteroendocrine cells and anterior pituitary cells (Roberts and Miyamoyto, 2015; Matschinsky and Wilson, 2019). HXKDC1 is only absent in muscle and adipose tissue, with high expression in colon, kidney and liver (Ludvik et al., 2016). ADPGK is highly expressed in immune cells of myeloid and lymphoid lineages (Imle et al., 2019).

**FIGURE 3:**
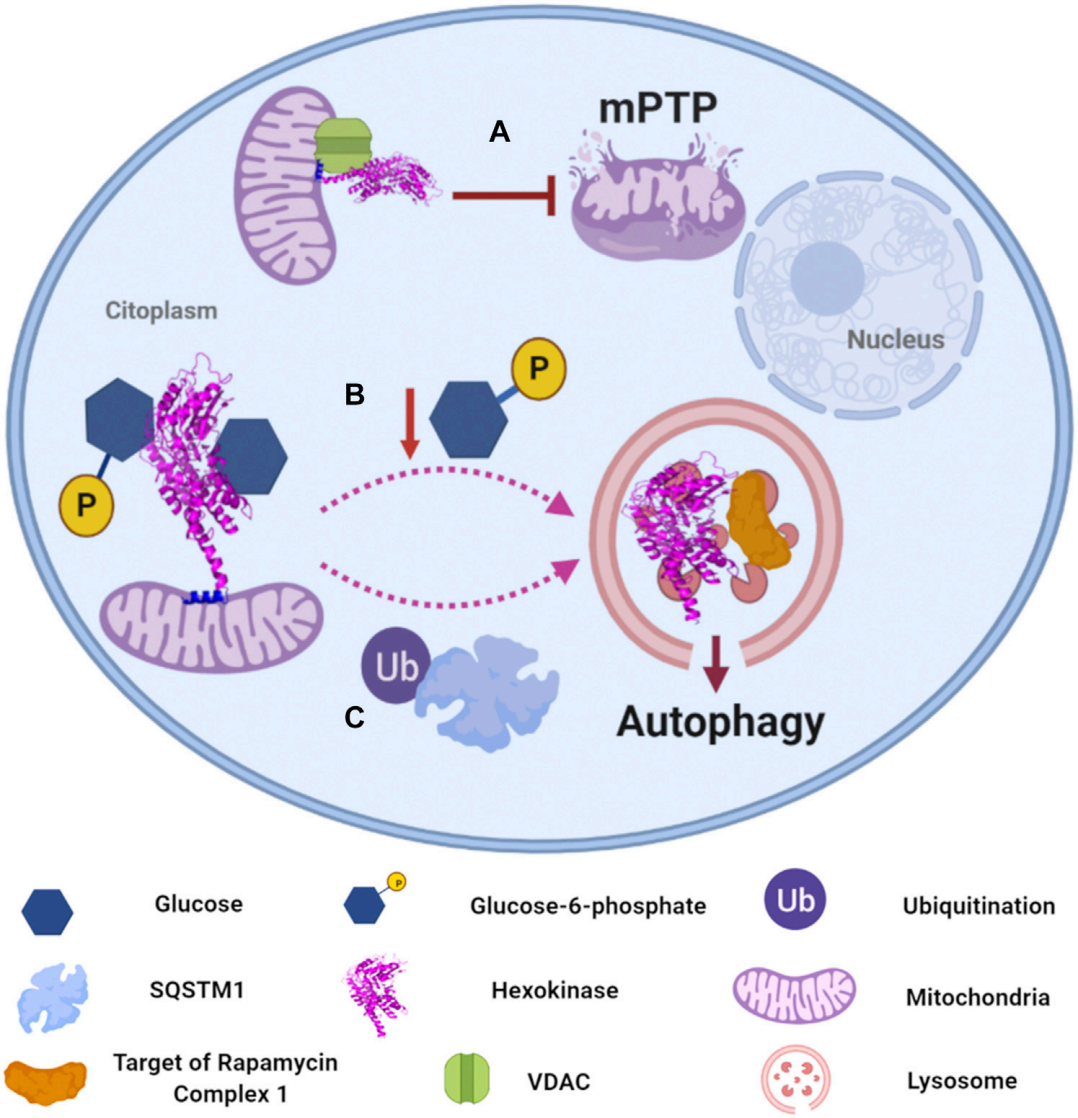
Human hexokinases moonlighting functions and regulation. **(A)** HXKII has a mitochondrial anchorage sequence that allows its association to function as a channel for glycolytic enzymes and mitochondrial ATP. Additionally, the association of HXKII to MOM inhibits the formation of the mitochondrial permeability transition pore (mPTP). (Arora and Pedersen, 1988; Baines et al., 2005; Mathupala et al., 2006). **(B)** Glucose-6-phosphate (G6P) mediates the regulation of autophagy. In its absence, HXKII can change its intracellular localization to regulate its association to TORC1, which may occur in lysosomes. The key factor responsible for activation/inactivation of autophagy is phosphorylated glucose (Betz and Hall, 2013; Kim et al., 2013a; Roberts et al., 2014). **(C)** Cells with high autophagic flux downregulate glycolysis by selectively degrading HXKII, which is Lys63 ubiquitinated to promote its recognition by the autophagy receptor SQSTM1, leading to its selective degradation in lysosomes (Jiao et al., 2018).

From all the human isoforms, HXKII has been extensively studied due to its implication in the progression and maintenance of tumor cells, hence other functions in which it is involved have been discovered. HXKI and HXKII have a mitochondrial anchorage sequence at their N-terminus that allows its association to the MOM and its interaction with the voltage-dependent anion channel (VDAC), the most abundant porin in MOM (Arora and Pedersen, 1988; Wilson, 2003). This association was demonstrated to be a “cellular strategy” to glycolytic enzymes and metabolites to gain “preferential access” to mitochondrial ATP, thus intimately coupling oxidative phosphorylation with carbon metabolism through ATP channeling from VDAC to HXKII (Mathupala et al., 2006). But HXKII association to MOM and VDAC offers an additional advantage: inhibiting the formation of mitochondrial permeability transition pore (mPTP) in cancer cells that overexpress HXKII and consequently, inhibiting apoptosis programmed cell death which is elicited by apoptotic Bcl-2 protein family and the release of cytochrome c through mPTP (Figure 3A). The entire inhibition mechanism is still to be elucidated, but there is evidence involving HXKII regulation. Furthermore, indirect HXKII interaction with cyclophilin-D, a mitochondrial matrix protein related to the opening of mPTP (Baines et al., 2005), seems to stabilize HXKII association to MOM, thus promoting a closed state of mPTP and providing protection to mitochondrial death pathways. In contrast, dissociation of HXKII from the mitochondria facilitates the formation of mPTP mediated by cyclophilin-D and promoting apoptosis (Machida et al., 2006; Roberts and Miyamoyto, 2015). Additionally, several reports have demonstrated that heterologous expression of VDAC can induce cell death, but the process can be mitigated by concomitant overexpression of HXKII (Pastorino and Hoek, 2008). In relation to this, an investigation showed that VDAC1, which is synthesized in cytosolic ribosomes, is found in both, cytosol and mitochondria, and cell death only occurs when the protein migrates to mitochondria. Translocation of VDAC1 seems to be regulated by HXKII protein levels, which apparently requires its catalytic activity, its N-terminal region, and the Glu73 residue to retain VDAC in the cytosol (Dubey et al., 2016). There is no information about the factors or signals involved in mitochondrial-translocation or cytosol-retention of the VDAC-HXKII complex, but it is likely to tilt the survival/death balance of the cell.

Furthermore, HXKII has been confirmed to mediate the regulation of autophagy in the absence of glucose-6-phosphate (G6P) (Roberts et al., 2014). Autophagy is a catabolic self-digestion system initiated by limited energy availability to ensure cellular energy homeostasis and survival. Overexpression of HXKII has been reported to promote autophagy, while its deletion inhibits the process even in the absence of glucose. So far, the elucidated mechanism involves HXKII binding to TORC1 (Target of Rapamycin Complex one), a negative regulator of autophagy, by its TOS (TOR signaling) motif, inactivating it (Roberts et al., 2014). According to experimental data, the key factor responsible for activation/inactivation of autophagy is the glucose phosphorylating activity, but not glucose binding (Figure 3B). However, the uncoupling between catabolic activity and autophagy regulation functions of HXKII is ambiguous since the experimental data did not show that the sugar-kinase activity of HXKII is independent of its G6P sensing function. It would require additional experiments, as mutating the HXKII catalytic site and determining whether it is still able to the allosteric recognition of G6P and autophagy prevention.

HXKII has also a role in the activation of the NOD-like receptor pyrin domain-containing 3 (NLRP3) inflammasome (Wolf et al., 2016), a cytosolic component of the innate immune system that regulates caspase-1 activation and secretion of proinflammatory cytokines IL-1β and IL-18 in response to cellular infections and cellular damage (Kelley et al., 2019). Wolf et al. (2016) identified that purified N-acetylglucosamine (NAG) or derived from degradation of bacterial peptidoglycan in phagosomes inactivate HXKII, leading to its dissociation from MOM and inducing inflammasome NLRP3 activation, indicating that HXKII is likely acting as a pattern recognition receptor. Interestingly detachment of HXKII from MOM can also be caused by the addition of G6P and jasmonate, a plant hormone (Goldin et al., 2008).

As discussed above, interaction of HXKII with VDAC in MOM allows HXKII to have preferential access to ATP, so inactivation and dissociation of HXKII from MOM implies a reduction in glycolytic flux and leads to interaction of VDAC to Bcl-protein family, which promotes opening of mPTP and subsequent apoptosis, so chemicals that induce this process could be used to reduce cancer cell proliferation, just as jasmonate does (Goldin et al., 2008). Moreover, metabolic disorders, such as diabetes or obesity, can directly induce inflammation (Wen et al., 2012) but it is still unclear whether the activation of NPLR3 by HXK follows the same mechanism in diabetes and obesity, as it does in inflammation caused by bacterial infection. Recently in the Pacific oyster *Crassostrea gigas*, it was described an HXK capable of recognizing various pathogen associated molecular patterns to activate an immune response against bacteria (Chen et al., 2021a), pointing out that the HXK receptor activity is necessary in different kingdoms. More research needs to be addressed to fully understand the different steps of the innate immune response mediated by the HXK and also to explore whether other organisms have a similar response against pathogens.

There is abundant information ascribing the role of GCK as a glucose sensor and single regulator of glucose homeostasis (Matschinsky and Wilson, 2019); however, GCK activity is important to the proposed Glc sensor activity, so it is difficult to determine if it is a moonlighting activity. Mice deficient in GCK (heterozygotes and homozygotes) and transgenic mice expressing GCK only in pancreatic β-cells were used to demonstrate that mice with only one GCK allele had elevated blood glucose levels and reduced insulin secretion, while *gck*-null mutants were lethal, and expression of GCK only in pancreatic β-cells was sufficient for survival. These experiments along with the investigation, description and screening of hundreds of nonsynonymous substitutions of GCK gene that causes inactivation or activation of GCK in many populations (Šimčíková et al., 2017) have been crucial to provide strong support to the hypothesis that GCK is a glucose sensor and a master regulator of glucose homeostasis (Grupe et al., 1995). Other experiments in mice provided detailed GCK links to diabetes mellitus and hypoglycemia diseases (Velho et al., 1992; Froguel et al., 1993; Grupe et al., 1995). There is also compelling evidence supporting the glucose-sensing function of GCK, and its role in glucose homeostasis in cells that participate in glucose homeostasis such as pancreatic α- and δ-cells, adrenal gland, glucose-sensitive neurons, enteroendocrine cells and anterior pituitary cells (Matschinsky and Wilson, 2019). Thus, GCK sensor activity in different cells seems to be vital to control the glucose homeostasis at the whole organism level and its mechanism of action is interconnected with the hormone production and secretion, so it is important to have a deeper understanding on how the sensor activity of GCK changes in different cells to provide strategies that might lead to manage the disorders associated with the low or high glucose blood levels.

Finally, for HXKI, Adams et al. (1991) showed evidence indicating that this hexokinase can also function as a protein-kinase capable of autophosphorylation and phosphorylation of histone 2A, solely in the absence of glucose, since when present, ATP is preferentially used for glucose-6-P production. Although there is still no physiological explanation for this protein-kinase activity, mammal HXKI is not the only hexokinase reported with this function: *S. cerevisiae* HXK2 have an autophosphorylation activity, which is stimulated *in vivo* by D-xylose (Fernández et al., 1988), the structural implications are discussed later. *Malus domestica* HXK1 has also been reported with a protein-kinase activity that apparently can lead to its self-phosphorylation *in vitro* and the participation of the protein in response to salt stress (Hu et al., 2016; Sun et al., 2018).

### Plants

**FIGURE 4:**
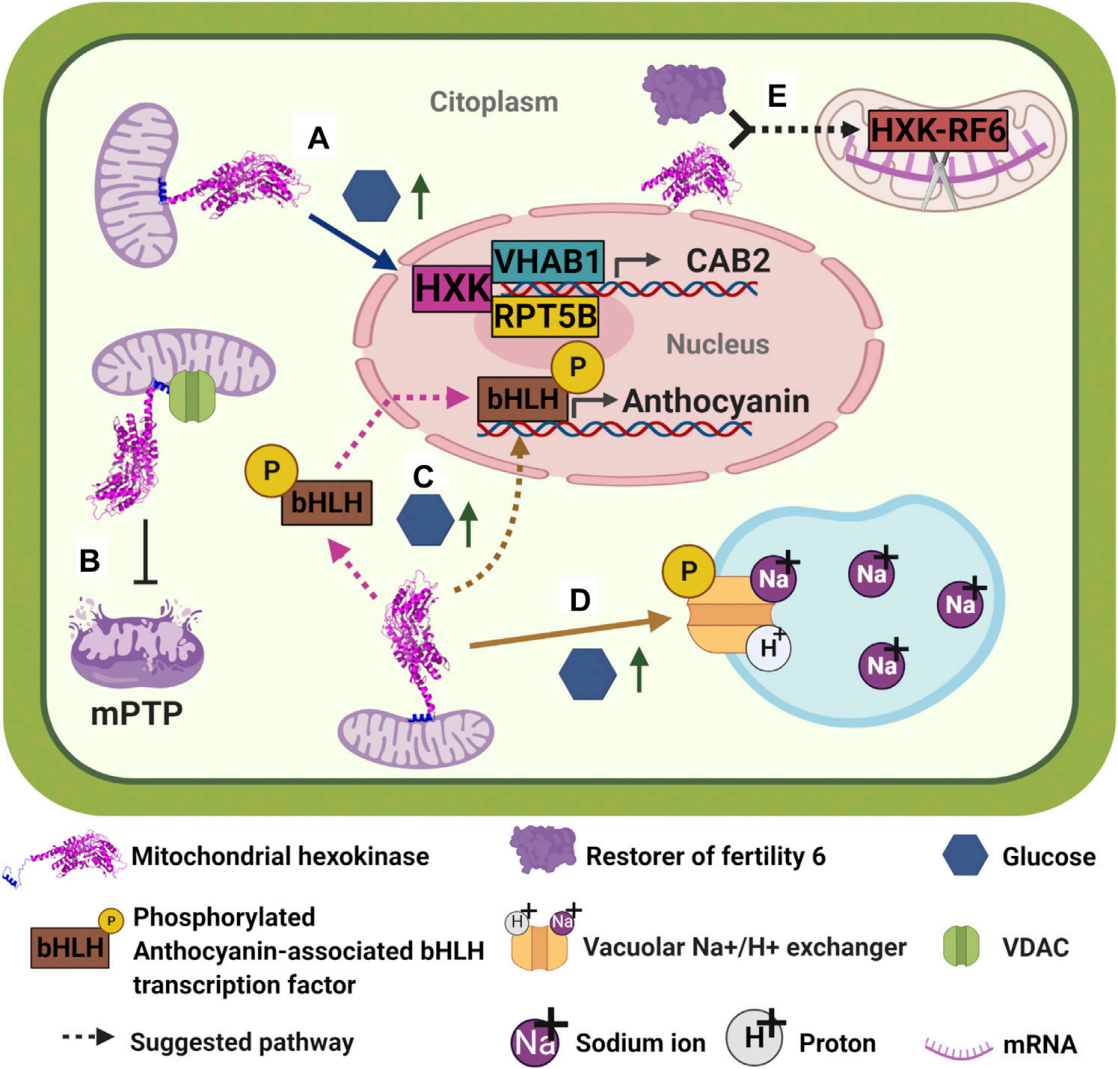
Plant hexokinases moonlighting functions. **(A)** At high glucose levels, AtHXK1 detaches from mitochondria and translocates to the nucleus in order to form a trimeric repressor complex with VHA-B1 and RPT5B; in turn, trimeric complex binds to putative transcription factors to bind on the *CAB2* promoter to repress transcription of CAB2 (Cho et al., 2006b). **(B)** It has been suggested that the association of plant HXKs to MOM occurs through interaction with VDAC. In turn, this interaction functions as an inhibitor of the formation of mPTP and subsequently of PCD (Kim et al., 2006; Godbole et al., 2013). **(C)** Apple MdHXK1 seems to be involved in the regulation of anthocyanins biosynthesis by interacting and phosphorylating an anthocyanin-associated bHLH transcription factor to stabilize it and enhance transcription of the anthocyanin biosynthesis genes, increasing anthocyanin production (Hu et al., 2016). It is not clear where the interaction between MdHXK1 and bHLH occurs: MdHXK1 is likely to interact and phosphorylate bHLH on cytoplasm and then phosphorylated-bHLH enters the nucleus to activate transcription of anthocyanin genes. However, it is also possible that MdHXK1 enters to the nucleus to interact with bHLH and phosphorylate it. None of the possibilities has yet been described. **(D)** MdHXK1 also contributes to glucose-mediated salinity tolerance by interacting and phosphorylating a vacuolar Na+/H+ exchanger (Sun et al., 2018). **(E)** *Oryza sativa* OsHXK6 helps restore the fertility of Honglian (HL) cytoplasmic male sterility (CMS). OsHXK6 and RF6 protein form a complex inside mitochondria which is capable of cleaving atp6-orfH79 transcript at nucleotide 1,238, avoiding orfH79 mRNA from its translation and therefore reestablishing pollen fertility (Huang et al., 2015). Similarly, OsHXK5 deficiency causes male sterility, however, the expression of a non-catalytic version of this protein, OsHXK5G113D, is not able to rescue the male-sterile phenotype (Lee et al., 2019).

In plants, hexokinases display a wide range of functions from glucose sensing to regulation of programmed cell death (PCD). *Arabidopsis thaliana*, for instance, expresses three catalytic hexokinases: mitochondrial hexokinases AtHXK1 and AtHXK2, and plastid AtHXK3 (Sarowar et al., 2008; Aguilera-Alvarado and Sánchez-Nieto, 2017). From the three of them, AtHXK1 has been widely studied for its additional function as a glucose sensor, in which it was demonstrated that at high glucose levels AtHXK1 is one of the key factors to repress photosynthetic gene transcription and arrest seedling growth (Moore et al., 2003). The mechanism involved has been partially elucidated: at high glucose levels, AtHXK1 form a trimeric repressor complex with the vacuolar H + -ATPase B1 (VHA-B1) and the 26S regulatory particle of proteasome subunit 5B (RPT5B); in turn, trimeric complex binds to putative transcription factors to bind on the CAB2 promoter to repress transcription of CAB2 (Figure 4A) (Cho et al., 2006b). It is unknown how AtHXK1 detaches from mitochondrial outer membrane and how AtHXK1, H + -ATPase B1, and RPT5B are translocated to the nucleus. However, it has been made clear that AtHXK1 renders its glucose-sensing function without needing its catalytic function, as observed by AtHXK1 catalytic mutants used to restore glucose-sensing phenotype on Arabidopsis null HXK1 mutants, *gin2-1* (Moore et al., 2003). Remarkably, *vha-B1* and *rpt5b* mutants display a glucose insensitive phenotype, even in the presence of AtHXK1, indicating that all three proteins are essential for glucose sensing and repression of photosynthetic genes (Cho et al., 2006b).

Kim and collaborators (2006) found that when *Nicotiana benthamiana* was silenced on its hexokinase 1, NbHXK1, necrotic lesions on leaves due to PCD were induced, suggesting a role of the HXK in the PCD (Figure 4B). On the other hand, Godbole and collaborators (2013) showed that overexpression of VDAC in tobacco cells protoplasts carried out PCD, but concomitant co-expression of mitochondrial hexokinase 3 from *N. benthamiana*, NbHXK3, rendered healthy protoplasts, suggesting a likely HXK-VDAC association in plant cells like that reported for mammals. This association between mitochondrial hexokinases and VDAC in plant cells has been hypothesized due to results found on beetroot cells, in which channeling of mitochondrial ATP to mitochondrial bound HXK through VDAC was evidenced (Alcántar-Aguirre et al., 2013). However, direct interaction between plant HXKs and VDAC remains to be demonstrated, and the mechanism involved in HXK-dependent programmed cell death pathway to be confirmed and described.

Additional functions of mitochondrial HXKs in plants have been described. Apple (*Malus domesticus*) mitochondrial hexokinase 1, MdHXK1, seems to be involved in both regulation of anthocyanins biosynthesis and glucose-mediated salt stress tolerance (Hu et al., 2016; Sun et al., 2018). Apparently, at high exogenous glucose levels, MdHXK1 is capable to interact and phosphorylate an anthocyanin-associated bHLH transcription factor at its Ser361 site, in order to stabilize it and thus enhancing transcription of the anthocyanin biosynthesis genes, thereby increasing anthocyanin production (Figure 4C) (Hu et al., 2016). Additionally, it was reported that the same MdHXK1 contributes to glucose-mediated salinity tolerance by interacting and phosphorylating a vacuolar Na^+^/H^+^ exchanger, MdHNX1, at its Ser275 site (Figure 4D). According to the authors, phosphorylation improved the stability of MdHNX1 and enhanced its Na^+^/H^+^ transport activity when MdHXK1 was overexpressed (Sun et al., 2018). Remarkably, assays utilizing catalytic mutants of MdHXK1 showed that both functions seem to be independent of its glucose-phosphorylation activity. No other plant HXKs have been reported to have protein phosphorylating activity, further investigation needs to be done to determine if other plant HXKs have that capacity.

In rice, besides the ability of OsHXK6 to sense glucose (Cho et al., 2009), this protein can process an mRNA transcript (Huang et al., 2015). To carry out this last function, OsHXK6 interacts with RF6 protein (restorer of fertility six); this interaction occurs between each protein C-terminus. Once inside the mitochondria, the complex OsHXK6/RF6 cleaves the atp6-orfH79 transcript at nucleotide 1,238, stopping orfH79 mRNA from being translated into protein (Figure 4E). orfH79 codes the ORFH79 protein, which interacts with a subunit of the mitochondrial complex III, impairing this complex activity and leading to pollen sterility. As no ORFH79 protein is synthesized, pollen becomes fertile again. RF6 and OsHXK6 are essential for processing the atp6-orfH79 transcript, but neither of them can bind directly to mRNA, and for this reason, additional proteins may be involved in this mRNA processing activity (Huang et al., 2015). Although OsHXK5 is also capable of rescuing the rice plant from pollen sterility, it may not follow the same mechanism that OsHXK6 does. The fact that pollen from plants lacking OshHXK5 show reduced hexokinase activity and the catalytic inactive protein, OsHXK5G113D, does not restore fertility, may indicate that this additional activity relies on the hexokinase activity rather than on the sugar sensing ability (Lee et al., 2019).

Finally, hexokinases 1, 2 and 3 from *Jatropha curcas* (JcHXK1, JcHXK2 and JcHXK3) seems to play a role in a different type of abiotic stress (Wang et al., 2019); qRT-PCR analysis showed that when 14-days old seedlings of *J. curcas* were exposed to low temperature during different periods of time, the levels of the three JcHXKs were upregulated. In this case, more studies need to be carried out to clearly determine the moonlighting role of these hexokinases.

## Regulation Mechanisms of Hexokinases’ Moonlighting Functions

The glucose phosphorylating activity of the HXK has been widely studied. Reviews on structural and enzyme kinetic features of HXKs are found in Cardenas et al., 1998 (HXKs from various organisms), Wilson, 2003 (mammal HXKs), Claeyssen and Rivoal, 2007 (plant HXKs) and Steitz et al., 1981 (yeast HXKs). It is known that mammal HXKI-III glucose phosphorylating activity are product-regulated: physiological concentrations of G6P can allosterically inhibit its activity in a non-competitive way with respect to glucose, but competitive to ATP (Van Schaftingen, 2013). In maize, mitochondrial attached HXKs are less inhibited by G6P and ADP than the cytosolic HXKs (Aguilera-Alvarado et al., 2019), suggesting that their subcellular localization is a key component to control its enzyme activity.

On the other hand, HXKs’ moonlighting activities are regulated by a wide range of mechanisms. A recent review analyzes the effect of redox state to change the function of diverse proteins in cancer cells, some of them are transformed in moonlighting proteins (Jiang et al., 2021). Understanding the existence of different multigene families with catalytic proteins, non-catalytic activities and pseudoenzymes which might appear to have different functions through evolution, can shed light on how a protein shows a different function depending on the cell context or the presence of some structural or motif differences (Jeffery, 2020). In this review, we collect different and varied examples that show how hexokinases are regulated in accord with different cellular localization, oligomeric state, phosphorylation/dephosphorylation state, concentration, and cell type to maintain cellular homeostasis and function.

### Transcriptional Regulation

In all living beings, to reduce the wasteful consumption of resources and energy a tight regulation of gene expression is crucial. For instance, in *S. cerevisiae* a complex regulation takes place to limit and specifically localize ScHXK2. In response to high glucose availability, ScHXK2 abundance increases and its localization changes. On glucose-supplemented medium, Mig1 sequesters ScHXK2 in the nucleus and the complex function as a gene repressor (Ahuatzi et al., 2004). Besides, the protein encoded by the *RGT*1 gene takes part in the glucose-induced expression of hexose transporter (HXT) genes (Ozcan et al., 1996), Rgt1 together with the Med8 protein represses the ScHXK2 gene expression when glucose is absent. This repression occurs when Rtg1 binds to a motif (CGGAAAA) located 395 bp upstream of the start codon in the ScHXK2 promoter (Palomino et al., 2005).

In mammals, as described earlier, HXKII has a role in tumorigenesis, since its overexpression provides a metabolic benefit and suppression of apoptosis, allowing cancer cells to grow indefinitely (Pastorino and Hoek, 2008). In cancer cells, expression of HXKII is regulated by several transcriptional regulators such as microRNAs, long non-coding RNA and some transcription factors (Ma et al., 2021). For example, Zheng et al. (2017) reviewed that HXKII expression is controlled by miR-155/miR-143 cascade in breast cancer, while in renal cell carcinoma, HXKII is co-regulated by miR-143/miR-451 and in hepatocellular carcinoma cells it is regulated by miR-199a. On the other hand, it has been reported that long non-coding RNA urothelial carcinoma associated 1(UCA1) overexpression promotes glycolysis by sequestering miR-203, which has an inhibitory effect on HXKII. This way, inhibition of miR-203 increases HXKII levels and, subsequently glycolysis rate (Liu et al., 2020). Finally, several transcription factors have been implicated in HXKII regulation in a variety of cancers, like hypoxia inducible factor 1α (HIF-1α) (Ma et al., 2021), the signal transducer and activator of transcription 3 (STAT3) and p53 (Rodríguez-Enríquez et al.,).

All these findings regarding the role of HXKII in cancer establishment and progression have been crucial to propose new therapeutic molecules that target this enzyme, as well as its regulators, to inhibit cancer cells proliferation. For instance, 3-bromopyruvate has been proved to break HXKII interaction with Apoptosis Inducing Factor (AIF) due to a covalent modification of mitochondria-bound HXKII (Wanga et al., 2009). On the other hand, methyl-jasmonate, a phytohormone related to defense mechanisms in plants, has been found to have anti-cancer properties due, in part, to its ability to remove HXKII from VDAC in apparently a selective manner. Detachment of HXKII from mitochondria triggers the reduction in glycolysis flux and mitochondrial membrane potential, which subsequently leads to the activation of apoptosis in cancer cells (Cohen and Flescher, 2009; Cesari et al., 2014).

### Posttranscriptional Regulation

Another way to regulate a gene expression is through posttranscriptional regulation mediated by AU-rich elements (AREs) (Shaw and Kamen, 1986) and ARE-binding proteins (Shyu and Wilkinson, 2000). Tristetraprolin (TTP), is a well-known ARE-binding protein able to promote the degradation of ARE-containing transcripts (Carballo et al., 1998; Chen et al., 2001; Lykke-Andersen and Wagner, 2005). In mammals, TTP is critical for the downregulation of HXKII expression in cancer cells as it enhances the degradation of the HXKII transcript, thus, suppressing the glycolytic capacity of cancer cells and reducing both the extracellular acidification rate and the oxygen consumption rate of cancer cells (Kim et al., 2019).

### Post-Translational Modifications

Post-translational modifications (PTMs) on moonlighting proteins allow the cells, to certain extent, to deal with environmental changes (Jeffery, 2016). It has been shown that several hexokinase functions are regulated by the phosphorylation-dephosphorylation process. As discussed earlier, in mammals, HXKII-mitochondria interaction provides protection to mitochondrial-mediated death pathways (Machida et al., 2006). HXKII, but not other isoforms contain a serine/threonine kinase Akt consensus sequence and a TOS motif, thus retaining two active kinase domains (Tsai and Wilson, 1996). Kinase Akt is required to inhibit apoptosis (Majewski et al., 2004) by phosphorylating HXKII at Thr473 (Roberts and Miyamoyto, 2015), leading to an increase of the association of HXKII to mitochondria and the reduction of its dissociation induced by G6P. Thr-473 phosphorylation does not seem to influence G6P binding to HXKII, but it does stabilize the HXKII-mitochondrial interaction (Roberts et al., 2013). It has also been reported that insulin treatment in adipose and skeletal muscle cell lines increases HXKII mRNA and protein levels through Akt pathways (Osawa et al., 1996; Culbert and Tavaré, 2002). The phosphorylation of hexokinase II by Akt is accompanied by increased binding of the enzyme to the mitochondria and contributes to the anti-apoptotic effects of Akt against ischemia/reperfusion injury in cardiomyocytes (Roberts et al., 2013). These observations illustrate the ability of Akt to positively regulate the binding of hexokinase II to VDAC (Pastorino and Hoek, 2008).

On the other hand, HXKII abundance can be regulated by degradation mediated by its ubiquitination in liver cancer cells. HXKII ubiquitinated on the Lys63 residue by the E3 ligase TRAF6 (TNF receptor-associated factor 6), thus promoting its recognition by the autophagy receptor sequestosome 1 (SQSTM1, a human protein ubiquitin-binding protein), leading to its selective degradation, and promoting the autophagy-mediated suppression of glycolysis (Figure 3C) (Jiao et al., 2018).

In *S. cerevisiae* phosphorylated ScHXK2 exists *in vitro* as two oligomeric states, a monomer, and a dimer (Furman and Neet, 1983). *In vivo*, the relative abundance of either the monomer or the dimer is affected by the phosphorylated state of ScHXK2. ScHXK2 has two sites for phosphorylation: one on serine-14, and this modification *in vivo* is inversely related to the extracellular glucose concentration (Kriegel et al., 1994), and another for serine-158 (Heidrich et al., 1997). Snf1 kinase is responsible for phosphorylating ScHXK2 at serine 14, while Glc7- Reg1 acts as a phosphatase (Ahuatzi et al., 2004; Vega et al., 2016). Glucose, and so does maltose and sucrose, are able to trigger the loss of the phosphate group from phosphorylated ScHXK2. Unlike these three sugars, raffinose, galactose, and ethanol trigger the appearance of both phosphorylated and dephosphorylated forms of ScHXK2. Even though the phosphorylated state of hexokinase is affected by the carbon source, it does not depend on glucose repression (Fernández-García et al.,).

ScHXK2 phosphorylation leads to: 1) Higher quantities of its monomeric form (Behlke et al., 1998), 2) increase of substrate affinity and inhibition by ATP (Golbik et al., 2001), and 3) stimulation of nucleocytoplasmic translocation of the phosphoenzyme (Fernández-García et al.,). Despite the findings made by Fernández-García et al. (2012) and collaborators regarding the phosphorylation site of ScHXK2, Kaps et al. (2015) found out that it is not serine-14, but serine 15 and that either Ymr291w or a Ymr291w-dependent protein are the responsible kinases. According to the authors, when yeast cells grow on a low glucose containing media, higher amounts of Ymr291w are detected, compared with a high-glucose one, therefore Ymr291w is likely to contribute to glucose signaling in a situation of limited external glucose availability. However, further investigation regarding the phosphorylation is needed to understand the structural and functional aspects of yeasts hexokinases *in vivo* phosphorylation.

When wild type *S. cerevisiae* cells are grown under repressing conditions, the Reg1-Glc7 complex dephosphorylates Mig1-ScHXK2, so that they can be imported into the nucleus, where ScHXK2 crosses the nuclear envelope by the α/β-importin (Kap60/Kap95) pathway (Peláez et al., 2012) and once inside forms a Mig1-ScHXK2 complex to recruit other proteins (Mig2, Snf1, Snf4, Gal83, and Reg1). Once all the proteins have been recruited, the expression of genes associated with growth on non-fermentable carbon sources, like *SUC2*, is repressed (Figure 2) (Moreno and Herrero, 2002; Ahuatzi et al., 2007). On the other hand, when *S. cerevisiae* cells are grown on low-glucose levels, the interaction between ScHXK2 and Mig1 is abolished by phosphorylation of ScHXK2 and Mig1 by Snf1 at serine 14 and serine 311, respectively (Ahuatzi et al., 2007; Fernández-García et al.,). Phosphorylation of both ScHXK2 and Mig1 results in their export from the nucleus to the cytoplasm by Xpo1 carrier protein (Peláez et al., 2009) and the subsequent disassembly of the repressor complex, allowing transcription of genes encoding metabolism proteins for non-fermentable carbon sources (Figure 2).

Nevertheless, the complex formation at the *SUC2* gene induced by ScHXK2 does not always depend on the phosphorylation state of ScHXK2, thus indicating that the phosphorylation state of ScHXK2 regulates only its location (nucleus or cytoplasm), but not its assembly and disassembly to the *SUC2* repressor complex (Vega et al., 2016). ScHXK2 assembly to the *SUC2* promoter may be regulated by conformational changes induced by glucose levels. As illustrated in Figures 1B,E, ScHXK2 is formed by the large and the small domains, separated by a deep cleft where the active site is located; when glucose is not present, ScHXK2 adopts an open, catalytically inactive conformation, but in the presence of glucose, the cleft closes due to a movement of both domains. Vega and collaborators (2016) found a correlation between ScHXK2 catalytically inactive conformation and its binding to the *SUC2* repressor complex, while an open conformation of ScHXK2 correlates with its dissociation from the *SUC2* promoter. With these findings, the authors suggest that the signaling activity of ScHXK2 is linked to conformational changes induced by glucose levels. However, it remains to be investigated if there are other factors or interactors in the process.

A distinct feature of some hexokinases that might contribute to its moonlighting activity is their subcellular location: some of them, like *S. cerevisiae* HXK2 (Fernández-García et al.,) and maize HXK7 and HXK8 (Aguilera-Alvarado et al., 2019) are located at the cytoplasm; others, in glycosomes, like *T. brucei* HXK2 (Blattner et al., 1995; Michaels et al., 2006); many in the mitochondrial outer membrane or MOM, like human HXKII (Arora and Pedersen, 1988; Dubey et al., 2016), *A. thaliana* HXK1 (Cho et al., 2006a) and apple HXK1 (Sun et al., 2018); or in the chloroplast, like *P. patens* HXK1 (Olsson et al., 2003) and *Oryza sativa* HXK4 (Cho et al., 2006a). And even there are HXKs that are translocated from one subcellular compartment to the nucleus, like AtHXK1 (Cho et al., 2006b) and ScHXK2 (Vega et al., 2016). The subcellular location depends partially on the presence of membrane anchor sequences, mitochondrial-targeting sequence (MTS), nuclear localization signals (NLS), or dual targeting signals (Cho et al., 2006b; Aguilera-Alvarado and Sánchez-Nieto, 2017). However post-translational modifications (PTM) might take place to change the localization of the protein from one compartment to the nucleus (Hashiguchi and Komatsu, 2017; Chen et al., 2021b), as occurs with ScHXK2. However, there are so many questions that need to be addressed to know the exact mechanism of the translocation process of various hexokinases to the nucleus, such as the type of PTM that takes place to change the protein destination. Hexokinase translocation between subcellular compartments, mainly to the nucleus, suggests that moonlighting proteins act as signaling intermediaries between organelles to promote rapid response to changes in the environment, substrate, or product concentrations and direct link between metabolic activity to genome integrity and gene expression (Kotiadis et al., 2014; Monaghan and Whitmarsh, 2015).

### Protein-Protein Interactions

As mentioned above, mammal HXKII attaches to MOM by interactions to VDAC and/or cyclophilin D, apparently as a strategy to couple oxidative phosphorylation with carbon metabolism through ATP channeling and to inhibit the formation of mPTP and subsequently, apoptosis programmed cell death (Figure 3A) (Mathupala et al., 2006; Pastorino and Hoek, 2008). Cyclophilin D, an immunophilin with *cis*-trans isomerase activity, affects mitochondrial binding of HXKII (Machida et al., 2006). Earlier studies have shown that the anti-apoptotic effects of cyclophilin D may be exerted by the stabilization of HXKII binding to mitochondria (Broekemeier et al., 1989). Inactivation of cyclophilin D with cyclosporine A or knock-down of its expression utilizing siRNA caused a release of mitochondrially bound HXKII. Moreover, the anti-apoptotic effects of cyclophilin D were overridden by the forced detachment of hexokinase II from the mitochondria. Such observations agree with the concept that HXKII prevents access of proapoptotic proteins such as Bax to the mitochondria or inhibits its pore-forming abilities. However, it is presently unclear how cyclophilin D activity regulates the binding of HXKII to the mitochondria (Pastorino and Hoek, 2008).

### Product Regulation

HXKII is required for the autophagy-mediated regulation of glycolysis, maintaining cellular homeostasis by modulating the level of glycolysis (Ros and Schulze, 2013), as mentioned earlier. HXKII and autophagy are linked through mTORC1 (Roberts et al., 2014); in cardiomyocytes, treated with 2-deoxy-D-glucose (2-DG), a non-metabolizable glucose analog, mTORC1 activity is decreased (Chantranupong and Sabatini, 2016), induction of autophagy is inhibited, and cell death induced by glucose deprivation is also increased (Tan and Miyamoto, 2016). Through its TOS motifs, HXKII binds to the Raptor domain of mTORC1 and this binding is largely increased by glucose absence (Schalm and Blenis, 2002; Nojima et al., 2003). Interestingly, HXK glycolytic and autophagic activities are regulated by G6P (Roberts et al., 2014); decreased levels of G6P promote HXKII-mTORC1 interaction and facilitate autophagy, while high levels of G6P inhibit HXKII-mTORC1 association and therefore cellular metabolism and growth can happen. This regulating function of HXKII occurs somewhere else but mitochondria, so it is likely that G6P changes intracellular localization of HXKII to regulate its association to mTORC1 (Figure 3B) (Roberts and Miyamoyto, 2015).

## What Remains to be Unraveled?

The available evidence from moonlighting proteins indicates that these moonlighting functions are not conserved across life domains, and the same happens with HXKs. The reported moonlighting activities are broad, from glucose sensor to hemoglobin binding protein, protein phosphorylating enzyme, immune receptor, apoptosis inhibitor and autophagy control regulator, among others. However, many of these proposed functions remain to be confirmed or need a deeper understanding.

As mentioned before, the glucose sensor activity is the most common moonlighting function of HXKs, though many aspects of the mechanism of sugar sensing in each kingdom need further investigation. Concerning ScHXK2, moonlighting functions are already known (Herrero et al., 1998; Randez-Gil et al., 1998; Ahuatzi et al., 2004; Fernández-García et al.,), but efforts are still to be made to demonstrate the signal perceived by ScHXK2 to assemble and disassemble the repressor complex in the *SUC2* promoter. If the conformational change in the ScHXK2 state is the signal needed to start the process (Vega et al., 2016) it will be a powerful tool to predict moonlighting proteins and their functions in the future.

It is important to emphasize that sugars are critical regulators of many processes in plants, such as germination, seedling development, photosynthesis, flowering, senescence, stress responses and carbon and nitrogen metabolism (Moore et al., 2003). It seems that moonlighting hexokinases may also be critical regulators in many of these processes. It is noticeable that it appears that moonlighting proteins are subservient to their “canonical” function so that they seem to take advantage of enzymatic activity or subcellular location to perform another function somehow related to the first one. In this way, plant mitochondrial HXKs seem to exploit their ability to phosphorylate glucose to sense it (or vice versa) and then generate a response to glucose levels that allow HXKs to translocate to the nucleus to repress or activate genes. However, it is unknown how AtHXK1 and its two partners are translocated from their original location to the nucleus to form the trimeric repressor complex (Cho et al., 2006b). It is possible that the complex is assembled inside the nucleus. If it is the case, it remains to know how the other two proteins in the complex perceive the glucose signal to start the translocation process to the nucleus.

Intriguingly, MdHXK1 upon high glucose levels activates anthocyanin biosynthesis genes (Hu et al., 2016) and increases the Na^+^/H^+^ transport activity at the vacuole to respond to saline stress (Sun et al., 2018). For these two additional functions described for MdHXK1, experiments need to be carried out to define the subcellular localization of MdHXK1-bHLH1 interaction to activate anthocyanin biosynthesis (Hu et al., 2016): does the interaction occurs in the cytoplasm and then both proteins are imported to the nucleus? Or does MdHXK1 translocates from the mitochondria to the nucleus and then interact and phosphorylates MdbHLH1? Furthermore, about MdHXK1 response to salt stress (Sun et al., 2018), what is the pathway involved in MdHXK1 migration to vacuole to interact with MdNHX1? What are the specific signals MdHXK1 perceives to perform one function or another specifically? Which cellular partners take part in each function so that the specific response the cell requires is generated?

Mitochondrial HXKs seem to display different functions during abiotic stress conditions, but in plants are likely to transduce also stress signals by pathogens, as overexpression of AtHXK1 and AtHXK2 induces PR genes expression (Sarowar et al., 2008).

Many reports regarding mitochondrial protein translocation to the nucleus, namely retrograde transport, exhibit nuclear localization upon cellular and environmental stimuli (Lionaki et al., 2016). Most of these dual-localization proteins contain a mitochondrial targeting sequence and a nuclear localization signal. However, some moonlighting proteins lack peptide signals; Cytochrome C is an example of it (González-Arzola et al., 2019). Due to lack of NLS, maybe cytochrome C could be exported to cytosol under non-apoptotic conditions, without mitochondrial membrane permeabilization, and then use a nuclear import mechanism by binding to nucleoporins (Jagot-Lacoussiere et al., 2015). Regarding plant mitochondrial HXKs, for several of them were reported the presence of an NLS within their amino acid sequence, adjacent to their MTS. Nevertheless, among species of higher plants as *Arabidopsis*, *Zea mays*, *Oryza sativa*, or the moss *Physcomitrella patens*, the organelle targeting signals could be ambiguous (Xu et al., 2013). For example, four hexokinases of moss *Physcomitrella patens*: PpHXK2, PpHXK3, PpHHXK7 and, PpHXK11 have an ambiguous targeting signal that leads to dual protein targeting to the outer membrane of mitochondria and the outer envelope of chloroplast (Nilsson et al., 2011; Xu et al., 2013). PpHXK4 contains a truncated N-terminus that differs from the plastid or mitochondrial hexokinases, and it is in the cytosol and nucleus. In *O. sativa*, only HXK7 and HXK8 are predicted with N-terminus truncated but do not resemble the PpHXK4 sequence (Nilsson et al., 2011).

Changes in the subcellular localization also can be due to post-transcriptional modifications related to the redox cellular state. Some proteins respond to the redox state, changing their subcellular localization. ROS and redox state affect the protein import machinery (Bölter et al., 2015; Ling and Jarvis, 2015), and also lead to changes in microtubule orientation in plant cells (Dang et al., 2018) and is well documented that in cancer cells low mitochondria activity promotes ROS production which affect several metabolic proteins including some glycolytic enzymes and moonlighting proteins, observations that are reviewed by Jiang et al. (2021). Redox post-transcriptional modifications seem to be site-specific by Cys residues and subject to temporal and spatial control (Foyer et al., 2020). Due to the perception of sugar signals or methyl-jasmonate related to mitochondrial ROS production, AtHXK1 could be translocated between the mitochondrion and nucleus (Claeyssen and Rivoal, 2007; Xiang et al., 2011), after being subject to redox modification, since there is a Cys-159 potentially oxidable near the HXK1 sugar-binding site (McConell et al., 2019). Finally, suggestions have been made around a glucose-induced conformational change, as suggested for ScHXK2 (Vega et al., 2016) or a variation on its oligomeric state (Moore, 2004).

Mammal HXKII is also a mitochondrial protein with non-canonical functions, like inhibition of apoptosis programmed cell death, demonstrated from research on cancer and neurodegeneration (Pastorino et al., 2002; Mathupala et al., 2006). Nevertheless, how HXKII and its different interaction partners modulate apoptosis in normal conditions is still a matter of investigation: which are the interaction partners and signals participating in the process? Where does HXKII-VDAC interaction occurs, and which signals modulate HXKII detachment from VDAC or MOM? On the other hand, regarding HXKII role in tumorigenesis, more investigation regarding the molecular signals and mechanisms that cause re-programing of HXKII to function aberrantly in cancer cells are needed in order to find compelling cancer therapeutics. It is not known if HXKII moonlighting ability is somehow related to its susceptibility to be re-programmed during cancer establishment; thus, keep digging on HXK moonlighting functions may shed light on the understanding of this possibility. As to HXKII regulation of autophagy, it is still unclear where HXKII translocates and what is the mechanism involved for this protein to interact with mTORC1 to activate the process. Is this function, in fact, a moonlighting function? Finally, referring to glucose homeostasis regulation by glucokinase (HXKIV), more data and experiments need to be carried out, as it is still a matter of discussion (Matschinsky and Wilson, 2019). Are suggestions around GCK as a single glucose sensor for glucose homeostasis regulation in mammals genuinely reliable? Does the system require additional factors for glucose sensitivity, as already discussed in plants?

## Concluding Remarks

HXKs have been extensively studied in mammals and yeasts. Nowadays, we have a significant amount of information to keep studying, analyzing, and investigating the impact of the moonlighting HXK functions in those species’ physiology. However, in plants, there is still a lack of information regarding not only hexokinases but moonlighting proteins in general. As sessile organisms, plants have been found to have large multigene protein families. Besides that, they have a complex phytohormone regulation and robust control of carbon and nitrogen sources. Thus, the presence of moonlighting proteins in plants may be more abundant and interconnected with different signaling pathways.

Thus, research is still needed to elucidate the regulatory mechanisms for the translocation of mitochondrial hexokinases to their different target organelles and to identify the pathway components and possible interaction partners that facilitate the responses to which plant mitochondrial hexokinases are associated.
